# Comparative Evaluation of Mogibacterium timidum in Diabetic Patients With Chronic Periodontitis, Nondiabetic Patients with Chronic Periodontitis, and Healthy Patients During Pretreatment and Posttreatment: A Real-Time PCR Study

**DOI:** 10.7759/cureus.72338

**Published:** 2024-10-24

**Authors:** Komal S Rajpurohit, Vidya M Dodwad, Pooja M Pharne, Neelam v Gavali, Nishita Bhosale, Yogesh Khadtare

**Affiliations:** 1 Department of Periodontology, Bharati Vidyapeeth (Deemed to be University) Dental College and Hospital, Pune, IND

**Keywords:** chronic periodontitis, comparative evaluation, diabetes and periodontitis, mogibacterium timidum, real time pcr

## Abstract

Introduction

*Mogibacterium* is a rod-shaped, anaerobic, gram-positive new bacteria. *Mogibacterium* genus included new species such as *Mogibacterium timidum*, *Mogibacterium vescum*, and *Mogibacterium pumilum* by taxonomic nomenclature in the year 2000. Its presence in oral bacterial flora is believed to increase severity of gingivitis and periodontitis, indicating some link between this bacterium and periodontal disease. Its frequency of detection in subjects with chronic periodontitis and those with diabetes along with chronic periodontitis has yet to be investigated. Therefore, the aim of this study was to determine the frequency of detection of *Mogibacterium timidum* in subgingival samples of such subjects.

Aims

We aimed to comparatively evaluate *Mogibacterium timidum* levels from subgingival plaque in non-diabetic subjects with chronic periodontitis, diabetic subjects with chronic periodontitis, and healthy subjects at baseline and six weeks post-scaling and root planing.

Materials and methods

The study was conducted in Bharati Vidyapeeth (Deemed to be University) Dental College and Hospital, Pune, India. A total sample size of 45 patients was chosen by first screening the patients on the basis of plaque index, probing pocket depth, and relative clinical attachment level. Subjects were divided into the following groups: group A comprised healthy subjects, group B comprised those with chronic periodontitis without diabetes, and group C comprised those with chronic periodontitis with diabetes. Subjects with probing pocket depth and clinical attachment level of 2-3mm were grouped into healthy subjects (group A). Subjects with pocket depth and clinical attachment level of more than or equal to 5mm were grouped into the chronic periodontitis group with and without diabetes in group B and group C, respectively. Real-time polymerase chain reaction was performed for plaque samples taken from gingival pockets for quantification and detection of the count of *M. timidum*.

Results

A statistical insignificant difference was found in the pre- and posttreatment counts of the bacteria in healthy subjects. A statistically significant difference was found in the pre- and posttreatment counts of DNA copies of *Mogibacterium timidum* in group B (chronic periodontitis subjects without diabetes) and group C (chronic periodontitis subjects with type 2 diabetes mellitus). The results indicate a significant association of this bacteria with periodontal destruction especially in diabetic subjects. The counts of the bacteria were highest in diabetic group, followed by the non-diabetic group (both having chronic periodontitis), with least counts in the healthy group both during pre- and posttreatment.

Conclusion

Significant association between chronic periodontitis, diabetes, and *Mogibacterium* is seen with the highest numbers of bacteria and destruction in the diabetic group, followed by the only chronic periodontitis group, and least association in the healthy group.

## Introduction

Periodontal disease causes progressive destruction of tooth-supporting structures through the interaction of bacteria with the host. Some conditions, such as apical periodontitis arising from endodontic lesions, may aggravate this destruction by forming endo-peri lesions [1]. Recent research has highlighted some undiscovered bacterial species involved in periodontal disease progression. It is possible to identify these microorganisms through novel laboratory investigations such as real-time polymerase chain reaction (PCR).

Pathogenic bacterial flora predominate mature plaque. *Mogibacterium timidum* was recently recognized as one of the many bacteria that form the host microbial flora in dental plaque. It is a rod-shaped, anaerobic, asaccharolytic, gram-positive bacteria identified in 2000. Mogibacterium is believed to increase the severity of gingivitis and periodontitis.

Evidence suggests that diabetes exacerbates periodontal disease and is a risk factor for periodontal disease progression [2,3]. Numerous studies have examined the differences in plaque composition between non-diabetic and diabetic subjects and found that diabetics have a higher quantity of periodontal pathogens [4,5]. Poor glycemic management and diabetes mellitus are therefore significant risk factors for periodontitis and increasing pathogenicity of subgingival microbiota [6,7]. According to these data, *Mogibacterium timidum* may be linked to periodontitis, and diabetics may be more susceptible to *Mogibacterium timidum* colonization in deep pockets. So far, no study has evaluated *Mogibacterium timidum* levels in diabetic subjects who also have chronic periodontitis. Thus, the goal of this research was to quantitatively analyze *Mogibacterium timidum* levels in subgingival plaque samples of subjects with periodontitis and uncontrolled type 2 diabetes and those with periodontitis but without diabetes.

The objectives of the study were to quantify *Mogibacterium timidum* in subgingival plaque samples from healthy subjects at baseline and six weeks post-scaling and root planing, to quantify *Mogibacterium timidum *species in subgingival plaque samples from non-diabetic patients with chronic periodontitis at baseline and six weeks post-scaling and root planing, to quantify *Mogibacterium timidum* species in subgingival plaque samples from diabetic patients with chronic periodontitis at baseline and six weeks post-scaling and root planing, and to comparatively quantify Mogibacterium in all three groups through real-time PCR at baseline and six weeks post-scaling and root planing.

## Materials and methods

Study protocol

The present study was conducted at Bharati Vidyapeeth (Deemed to be University) Dental College and Hospital, Pune, India, and the procedures followed were in accordance with the ethical standards established by the Institutional Ethics Committee (IEC) with IRB no. EC/NEW/INST/2021/MH/0029.

Inclusion criteria

Subjects ready to sign written informed consent forms, subjects above 30 years of age; subjects willing to adhere to the study protocol, systemically healthy subjects (exception: diabetes mellitus), subjects who were diabetic for the last five years, subjects with at least 30% sites having probing depth and clinical attachment level (CAL) ≥ 5mm, subjects with glycemic status (a single laboratory analyzed the glycated hemoglobin for diabetic subjects), and subjects with HbA1c values greater than 7% were included in the study.

Exclusion criteria

Subjects with systemic illnesses, subjects who underwent surgical therapy in the last six months, pregnant women and breast-feeding mothers, subjects who underwent antibiotic therapy in the previous six months, subjects who smoke and have smoked within the last five years, tobacco chewers, subjects who received periodontal and antimicrobial therapy in the last six months, subjects who used antimicrobial mouthwashes in the last three months, subjects having any chronic systemic disease (exception: diabetes mellitus) that could affect periodontal tissues, subjects with prolonged use of immunosuppressive medication, and subjects suffering from major vascular complications of diabetes were excluded.

Sample size was determined by statistical analysis using SPSS Version 20 (IBM Corp., Armonk, NY). According to the selection criteria, subjects were grouped into healthy subjects (group A), chronic periodontitis subjects without diabetes (group B), and chronic periodontitis subjects with diabetes (group C). A total of 45 individuals visiting Bharati Vidyapeeth Dental College were included in the study. Relevant case histories, including the chief complaint and medical and dental histories, were detailed in a specific format. Periodontal clinical examination was conducted with the necessary periodontal armamentarium, including the University of North Carolina Probe (UNC 15). After providing the subjects with the details of the study, written consent was obtained from them.

The participants’ periodontal status was evaluated using the following parameters: Plaque Index (Turesky-Gilmore-Glickman modification of the Quigley Hein 1978), probing pocket depth, and relative CAL.

After evaluating these parameters, the subjects were divided into three groups (Table 1): group A comprised 15 healthy subjects, group B comprised 15 subjects with chronic periodontitis that did not present with type 2 diabetes mellitus, and group C comprised 15 subjects with chronic periodontitis that presented with type 2 diabetes mellitus for at least the last five years.

**Table 1:** Grouping subjects based on plaque index, probing depth, and relative clinical attachment level Plaque index of 0-1, pocket probing depth, and relative CAL of 2-3mm were grouped as healthy subjects. Plaque index of >2mm, pocket probing depth >5mm, and relative CAL >5mm were categorized as group B. Group B patients were non-diabetic. Plaque index of >2mm, pocket probing depth, and relative CAL of >5mm were categorized as group C. Group C patients were diabetic since the past five years, with HbA1c values above 7% from the past five years.

	Group A (healthy)	Group B (chronic periodontitis patients without diabetes)	Group C (chronic periodontitis patients with diabetes)
Plaque index (Turesky-Gilmore -Glickman modification of Quigley Hein 1978)	0-1	>2	>2
Pocket probing depth	2-3mm	>5mm	>5mm
Relative clinical attachment level	2-3mm	>5mm	>5mm

Collection of subgingival plaque samples

The selected sites were isolated with cotton rolls. To avoid contamination prior to sampling, sterile cotton pellets were moved around the tooth to remove supragingival plaque. Samples of subgingival plaque were collected from periodontal pockets before scaling and root planing using sterile Gracey curettes in a similar manner as stated in the study by Shikarkhane et al. [8]. The samples were transported in tris-EDTA medium after labelling them with the group number and sample number to Marath Mandal Dental College within 48 hours of collection. The bacterial count of Mogibacterium timidum was evaluated using the real-time PCR technique. Immediately after sample collection, thorough scaling and root planing were performed. Six weeks after scaling and root planing, subgingival plaque samples were again collected to analyze the same bacterial count.

In vitro analyses

Subgingival plaque samples that were transported to the laboratory were collected in 1.5 mL microcentrifuge tubes containing tris-EDTA buffer (TE buffer). The samples were then processed for extraction of DNA using the proteinase K method (modified).

DNA extraction

Plaque samples were washed with fresh TE buffer thrice, followed by the addition of 50 µL of lysis buffer I (1% Triton X-100, Tris-HCL pH 8.0 10 mM, and EDTA 1 mM) and then 50 μL of lysis buffer II (Tris-HCl pH 8.0 50 mM, KCL 50 mM, MgCl2 2.5 mM, Tween-20 0.45%, and Nonidet P-40 0.45%). Proteinase K (10mg/mL) was added to degrade the protein. The samples were incubated for 2 hours at 60°C and then for 10 minutes in a boiling water bath. DNA purification was done using absolute ethyl alcohol and 3M sodium acetate. Molecular-grade water at -20°C was used to dissolve the DNA pellet. The quality and purity of DNA samples were confirmed by using a BioPhotometer (Eppendorf, Hamburg, Germany) [9].

Real-time PCR

A RealPlex Mastercycler (Eppendorf, Hamburg, Germany) was used to perform real-time qPCR amplification and detection with the aid of a 96-well format. A laminar airflow hood was used to limit contamination. Primers targeting the species-specific sequence of the 16s ribosomal ribonucleic acid gene of Mogibacterium timidum were used in the study: forward primer 5ʹ- AAGCTTGGAAATGACGC-3ʹ and reverse primer 5ʹ- CCTTGCGCTTAGGTAA-3ʹ.

The reaction was performed in a total volume of 20 µL in 0.2 mL of clear-cap tube strips. TB Green Premix Ex Taq (Tli RNaseH Plus) PCR master mix was used in the reaction mixture, which contained TaKaRa Ex Taq HS, dNTP mixture, Mg2+, Tli RNase H, and TB green dye. DNA templates and primers were added at optimum concentrations. The tubes were then kept in a real-time PCR Mastercycler (Eppendorf, Hamburg, Germany) to run different temperature cycles.

After enzyme activation for 3 minutes at 95°C, denaturation of 40 repeats was performed for 20 seconds at 95°C. The time period and temperature for annealing were 20 seconds and 50-60°C, respectively. The extension was conducted for 20 seconds at 72°C [10].

Standard curve and analysis

To generate the standard curve, real-time PCR was performed with serially diluted samples of the standard DNA samples (ATCC No. 33093). Deionized water served as a negative control. The PCR was optimized with standard DNA samples with a slope of -3.2, R2 value of 0.997, and efficiency of 1.05.

The graph of the amplification plot depicts fluorescence versus cycle numbers. The Ct value was identified based on the number of cycles that were needed for the fluorescent signal to cross the threshold level. The amount of target nucleic acid in the sample is inversely proportional to the Ct value. Using the Ct values of standard DNA samples, a standard curve was plotted. Ct values were plotted by running unknown samples in real-time PCR to obtain the Ct values for each sample.

Plan of statistical analyses

All the statistical analyses were performed using SPSS Version 20 (IBM Corp., Armonk, NY) for qualitative and quantitative data.

## Results

**Figure 1:**
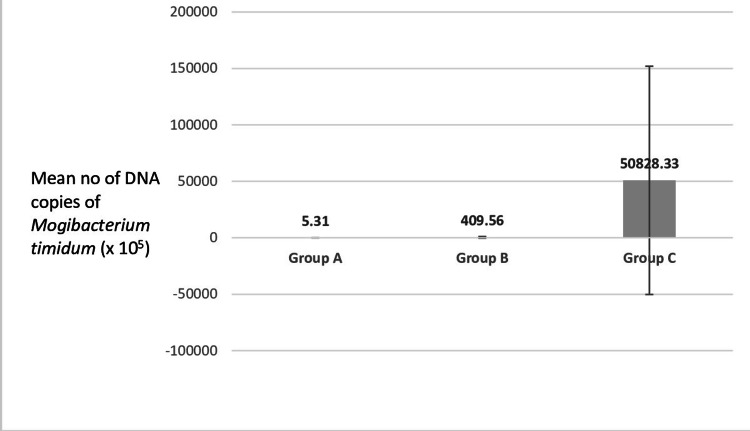
Intergroup comparison of pretreatment DNA copies of Mogibacterium timidum between the three study groups The highest number of *Mogibacterium timidum* copies were found in group C (diabetic patients with chronic periodontitis) with 50,828.33 x 10^5^, followed by group B (chronic periodontitis patients without diabetes) with 409.56 x 10^5^. The healthy group showed the minimum number of DNA copies, indicating the least presence of this bacterium. This significant difference between the three groups highlights the fact that *Mogibacterium* directly influences the severity of periodontal disease.

Intergroup comparison of pretreatment DNA copies of *Mogibacterium timidum* between the three study groups showed that the highest number of *Mogibacterium timidum* copies were found in group C (diabetic patients with chronic periodontitis) with 50,828.33 x 10^5^, followed by group B (chronic periodontitis patients without diabetes) with 409.56 x 10^5^. The healthy group showed the minimum number of DNA copies, indicating the least presence of this bacterium. This significant difference between the three groups highlights the fact that *Mogibacterium* directly influences the severity of periodontal disease (Figure 1, Table 2).

**Table 2:** Intergroup comparison of pretreatment DNA copies of Mogibacterium timidum between the three study groups *A p-value of ≤0.05 is statistically significant The mean number of DNA copies was 2.46 x 10^5^ million copies in group A, 409.56 x 10^5^ in group B, and 50,828.33 x 10^5^ in group C. The standard deviation was 16.69, 762.94, and 101,099.00 in group A, group B, and group C, respectively. The p-value was <0.001, which was statistically significant.

No. of DNA copies	Group A	Group B	Group C
Mean (x10^5^)	2.46	409.56	50,828.33
Standard deviation (x10^5^)	16.69	762.94	101,099.00
Kruskal-Wallis ANOVA test	<0.001*		

**Figure 2:**
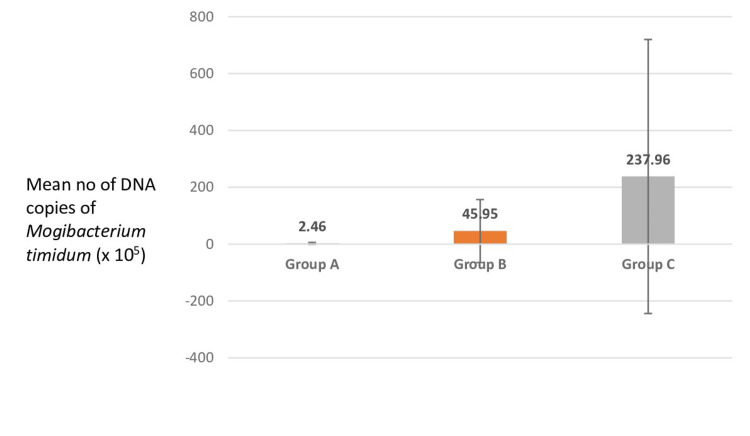
Intergroup comparison of posttreatment DNA copies of Mogibacterium timidum between the three study groups Post-scaling and polishing, the highest number of DNA copies of *Mogibacterium timidum *was present in group C (diabetic patients with chronic periodontitis) with 237.96 x 10^5^, followed by group B (chronic periodontitis patients without diabetes) with 45.95 x 10^5^. The healthy group again showed the minimum number of DNA copies of 2.46 x 10^5^, indicating the least presence of this bacterium in health. This difference is insignificant in indicating the elimination of bacteria post-scaling.

Intergroup comparison of posttreatment DNA copies of *Mogibacterium timidum* between the three study groups showed that the highest number of DNA copies of *Mogibacterium timidum* was present in group C (diabetic patients with chronic periodontitis) with 237.96 x 10^5^, followed by group B (chronic periodontitis patients without diabetes) with 45.95 x 10^5^. The healthy group again showed the minimum number of DNA copies 2.46 x 10^5^, indicating the least presence of this bacterium in health. This difference is insignificant in indicating the elimination of bacteria post-scaling (Figure2, Table 3).

**Table 3:** Intergroup comparison of posttreatment DNA copies of Mogibacterium timidum between the three study groups The mean number of DNA copies was 2.46 x 10^5^ million in group A, 45.95 x 10^5^ in group B, and 237.96 x 10^5^ million in group C. The standard deviation was also 3.74 in group A, 110.85 in group B, and 482.07 in group C. The p-value was <0.005, which was not statistically significant.

No. of DNA copies	Group A	Group B	Group C
Mean (x10^5^)	2.46	45.95	237.96
Standard deviation (x10^5^)	3.74	110.85	482.07
Kruskal-Wallis ANOVA test	0.184		

**Figure 3:**
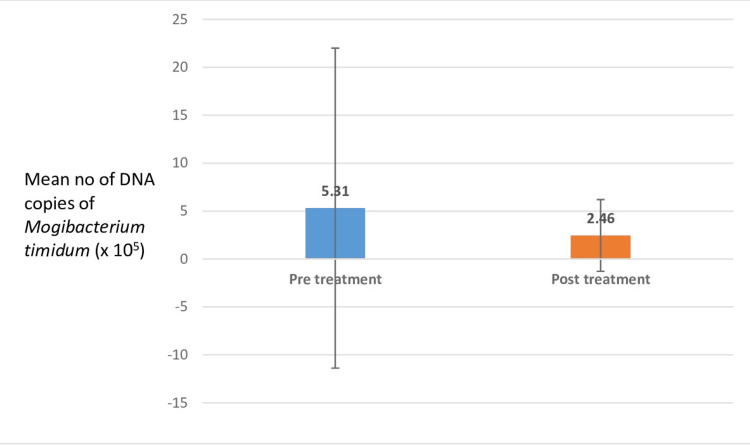
Comparison of pre- and posttreatment count of DNA copies of Mogibacterium timidum in group A (healthy patients) The comparison of the number of DNA copies of* Mogibacterium timidum* in group A (healthy subjects) showed 5.31 x 10^5^ million during pretreatment and 2.46 x 10^5^ million posttreatment, indicating a statistically significant difference and minor numbers of this bacterium in healthy subjects.

The comparison of the number of DNA copies of *Mogibacterium timidum* in group A (healthy subjects) showed 5.31 x 10^5^ million during pretreatment and 2.46 x 10^5^ million posttreatment, indicating a statistically insignificant difference and minor numbers of this bacterium in health (Figure 3, Table 4).

**Table 4:** Comparison of pre- and posttreatment count of DNA copies of Mogibacterium timidum in group A (healthy patients) The mean value of the DNA copies in group A during pre- and posttreatment were 5.31 x 10^5^ and 2.46 x 10^5^, respectively. The standard deviation during pre- and posttreatment in group A was 16.69 and 3.74, respectively. This does not show statistical significance.

No. of DNA copies	Pretreatment	Posttreatment
Mean (x10^5^)	5.31	2.46
Standard deviation (x10^5^)	16.69	3.74
Wilcoxon signed-rank test	0.433	

**Figure 4:**
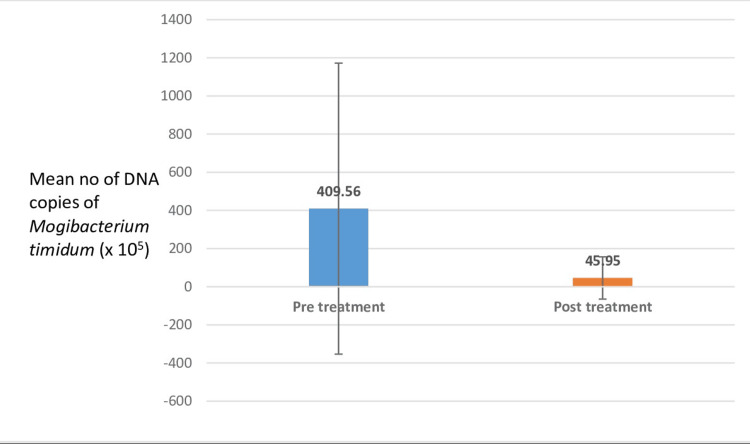
Comparison of pre- and posttreatment count of DNA copies of Mogibacterium timidum in group B (chronic periodontitis patients) The mean values of the DNA copies in group B during pre- and posttreatment were 409.56 x 10^5^ and 45.95 x 10^5^, respectively, with a standard deviation of 762.94 and 110.85, respectively. This result shows a statistically significant difference with a p-value of <0.05, indicating a direct correlation of this bacterium with periodontal destruction.

The mean values of the DNA copies in group B during pre- and posttreatment were 409.56 x 10^5^ and 45.95 x 10^5^, respectively This result shows a statistically significant difference with a p-value of <0.05, indicating a direct correlation of this bacterium with periodontal destruction (Figure 4, Table 5).

**Table 5:** Comparison of pre- and posttreatment count of DNA copies of Mogibacterium timidum in group B (chronic periodontitis patients) The mean value of the DNA copies in group B during pre- and posttreatment were 409.56 x 10^5^ and 45.95 x 10^5^, respectively, with a standard deviation of 762.94 and 110.85 during pre- and posttreatment, respectively. *A p-value of <0.05 indicates a statistically significant difference.

No. of DNA copies	Pretreatment	posttreatment
Mean (x10^5^)	409.56	45.95
Standard deviation (x10^5^)	762.94	110.85
Wilcoxon signed-rank test	0.030*	

**Figure 5:**
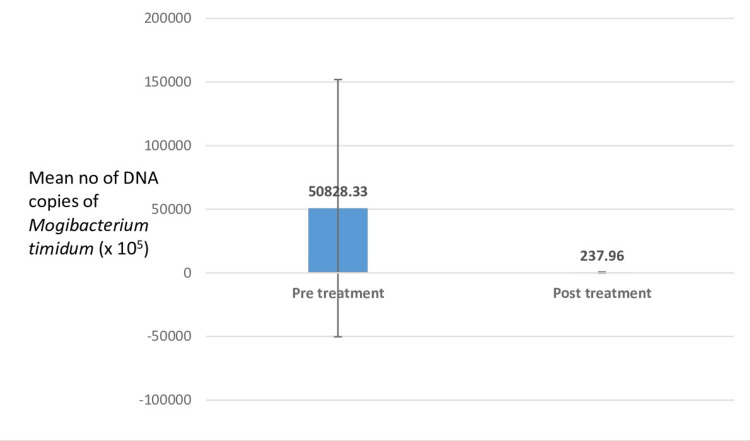
Comparison of pre- and posttreatment count of DNA copies of Mogibacterium timidum in group C (chronic periodontitis with diabetes patients) The number of DNA copies of *Mogibacterium timidum* in group C was 50,828.33 x 10^5^ million pretreatment and 237.96 x 10^5^ million posttreatment, which shows a statistically significant difference, again indicating a direct correlation with periodontal destruction. The values showed a significant decline during pre- and posttreatment in both diabetic and non-diabetic groups, indicating a definite correlation of this bacterium with periodontal destruction. Moreover, the bacterial count was higher in the diabetic group than in the non-diabetic group, indicating a direct correlation with periodontal destruction.

Comparison of pre- and posttreatment count of DNA copies of *Mogibacterium timidum* in group C showed that the number of DNA copies was 50,828.33 x 10^5^ million pretreatment and 237.96 x 10^5^ million posttreatment. This shows a statistically significant difference, again indicating a direct correlation with periodontal destruction (Figure 5, Table 6).

**Table 6:** Comparison of pre- and posttreatment count of DNA copies of Mogibacterium timidum in group C (chronic periodontitis with diabetes patients) The mean value of the DNA copies in group B during pre- and posttreatment were 50,828.33 x 10^5^ and 237.96 x 10^5^, respectively, with a standard deviation of 101,099.00 and 482.07 during pre- and posttreatment, respectively *A p-value of <0.05 indicates a statistically significant difference.

No. of DNA copies	Pretreatment	posttreatment
Mean (x10^5^)	50,828.33	237.96
Standard deviation (x10^5^)	101,099.00	482.07
Wilcoxon signed-rank test	0.002*	

All the values showed a significant decline posttreatment in both diabetic and non-diabetic groups, indicating a definite correlation of this bacterium with periodontal destruction. Moreover, the bacterial count was higher in the diabetic group than in the non-diabetic group, indicating a direct correlation with periodontal destruction.

## Discussion

Different microbes naturally present in the oral cavity, along with poor oral hygiene practices, cause dysbiotic bacterial flora, which leads to periodontal disease. Gram-negative bacteria, such as *Campylobacter rectus*, *Aggregatibacter actinomycetemcomitans*, *Prevotella intermedia*, *Treponema denticola*, and *Porphyromonas gingivalis*, and gram-positive bacteria, such as *Parvimonas micra* and *Eubacterium timidum*, are the most common complex bacterial species that contribute to this disease.

A new genus named *Mogibacterium timidum *was discovered in the oral microbiota in 2000. It is rod-shaped, gram-positive, asaccharolytic, strictly anaerobic, non-motile, and non-spore-forming in nature. Its existence in the oral microbiota plays a role in infectious oral illnesses. Studies have shown that the presence of *Mogibacterium timidum* in higher numbers demonstrates increased severity of gingivitis [11,12]. Moore et al. [13] revealed the existence of *Mogibacterium timidum* in periodontal pockets that show signs of both "juvenile" and chronic periodontitis. This bacterium is also connected to several kinds of infections in the craniofacial region. A study evaluated the microbe diversity of acute lung and liver infections and abscesses in the head and neck region and found *Mogibacterium timidum* in multiple affected regions through culture and biochemical techniques [14].

Compared to numerous studies that have investigated the important role of inflammation in relation to diabetes and periodontitis, relatively few studies have analyzed the relationship between the oral microbiota and diabetes.

In a study involving patients with type 1 diabetes mellitus in the Japanese population, P. gingivalis and P. intermedia were discovered in higher concentrations in diabetic subjects with chronic periodontitis than in periodontally healthy subjects [15]. These results imply that there might be slight variations in the microbiota constituents of subgingival areas between patients with and without diabetes.

Adolescents with diabetes exhibited changes in the anaerobic growth of gram-negative bacteria and the subgingival microbial flora, with the highest detection frequency observed in diabetics with inadequate glycemic control. Compared to well-managed diabetic participants, those with poorly controlled diabetes exhibit increased attachment and alveolar bone loss, indicating the presence of these microbes in associated plaque.

These effects occur because diabetes affects the surrounding conditions in the periodontal pocket in a way that promotes the development of specific species of bacteria. Despite *Mogibacterium timidum* being demonstrated as an important bacterium for periodontal disease, no research has yet examined the frequency of identifying this bacterium in individuals with diabetics versus those without the disease in chronic periodontitis. Furthermore, no prior assessment has been conducted on the *Mogibacterium timidum* levels in diabetic subjects with chronic periodontitis [16].

Moreover, considerable evidence suggests that periodontitis has negative effects on glucose regulation and that diabetes worsens periodontitis [17,18]. According to theories, diabetes mellitus raises the risk of periodontitis through nonenzymatic pathways that result in advanced glycation end products, such as tumor necrosis factor and IL-1, hindering the activity of proinflammatory cytokines and elevating toll-like receptor expression [19]. Other potential reasons include reduced blood flow in capillaries and recurrent infections due to diminished polymorphonuclear function in diabetic subjects.

Renato Corrêa Viana Casarin conducted a study in 2012 to determine the incidence of identification of *Mogibacterium timidum *in subgingival samples obtained from patients with uncontrolled diabetes and non-diabetics with generalized chronic periodontitis and generalized aggressive periodontitis [20]. In this trial, 48 subjects had generalized aggressive periodontitis, 50 were non-diabetic adults, and 39 subjects had uncontrolled type 2 diabetes (glycated hemoglobin > 7%) and generalized chronic periodontitis. When a pocket was probed deeper than 7mm, subgingival biofilm was collected. Using PCR and subsequent DNA extraction, *Mogibacterium timidum *was identified, and outcomes were analyzed using the chi-square test. The results showed higher numbers of bacteria in diabetic individuals, indicating a difference in the plaque microbial composition of diabetics versus non-diabetic subjects.

In our study, a statistically significant difference was noted in the intergroup comparison of pretreatment DNA copies of *Mogibacterium timidum*, indicating the highest numbers in chronic periodontitis subjects with diabetes, followed by chronic periodontitis subjects, and negligible numbers in healthy subjects. A statistically insignificant difference in the pretreatment and posttreatment counts of DNA copies of *Mogibacterium timidum* in group A (healthy patients) indicates that this bacterium is a minor component in periodontally healthy subjects. A statistically significant difference in the pretreatment and posttreatment counts of DNA copies of *Mogibacterium timidum* in group B (chronic periodontitis patients) indicates its association with the dysbiotic microbiota of chronic periodontitis. Also, a statistically significant difference in comparison of pre- and posttreatment counts of DNA copies of *Mogibacterium timidum *in Group C (chronic periodontitis patients with diabetes) demonstrates that this bacterium is associated with periodontal breakdown. Finally, a statistically insignificant difference in the intergroup comparison of posttreatment DNA copies of *Mogibacterium timidum* between the three study groups indicates post-intervention elimination of this bacterium from subgingival plaque. In our study, although this bacterium is found in healthy subjects, the numbers are relatively insignificant in causing disease compared to the other pathogenic microbes. We also found the highest number of these bacteria in diabetic individuals, in accordance with the studies mentioned above.

Increased local inflammation and poor glucose control in diabetics may alter the subgingival microbial profile as well as the subgingival environment and lead to advanced-stage disease. Analyzing the microbial count of *Mogibacterium timidum* in our current investigation leads us to conclude that increased bone loss, attachment loss, and periodontal breakdown are somehow associated with the rise in *Mogibacterium timidum *in the subgingival flora in association with chronic periodontitis; moreover, they are more highly associated in diabetics with chronic periodontitis. Thus, when studying specific microbes in the subgingival biofilm, diabetic subjects seem crucial due to their connection with the development of severe periodontitis [21,22]. The higher incidence of periodontitis in diabetic individuals is conclusively associated with the microbial composition of dental plaque, with Mogibacterium being one of them.

## Conclusions

The limitation of this investigation is that since *Mogibacterium* is a new bacterium, it cannot be confirmed that it is a definite risk indicator for periodontal breakdown. Secondly, the virulence factors related to this bacterium have not been discovered yet. Also, the pathogenic pathway of destruction of periodontal disease in relation to this bacterium requires further investigation. Although the counts are higher in disease states, the pathogenicity of this bacterium is questionable. Further investigations confirming the presence of *Mogibacterium* in periodontal disease and its virulence factors are needed to support the current investigation. Therefore, in order to ascertain this species’ true contribution to periodontal degradation and to formulate a strategy for controlling its infection, future studies should consider its virulence factors and pathogen-associated molecular patterns. To conclude, *Mogibacterium timidum* has a significant role in intensifying inflammation-related responses in oral tissues in individuals presenting with diabetes mellitus.

A definite association between *Mogibacterium* and periodontitis is seen in the current investigation. Also, increase in the number of this bacterium is seen in diabetics with periodontal disease. However, more than 400 species of bacteria affect periodontal disease. *Mogibacterium* species may have an impact on the deterioration of periodontal tissues, and in light of the current study's findings, *Mogibacterium timidum *should be factored into subsequent research. Future research ought to consider these findings and determine the potential pathogenic route and virulence factors associated with *Mogibacterium timidum*.

## References

[1] Kato T, Fujiwara N, Kuraji R, Numabe Y (2020). Relationship between periodontal parameters and non-vital pulp in dental clinic patients: a cross-sectional study. BMC Oral Health.

[2] Grossi SG, Genco RJ (1998). Periodontal disease and diabetes mellitus: a two-way relationship. Ann Periodontol.

[3] Papapanou PN (1996). Periodontal diseases: epidemiology. Ann Periodontol.

[4] Mandell RL, Dirienzo J, Kent R, Joshipura K, Haber J (1992). Microbiology of healthy and diseased periodontal sites in poorly controlled insulin dependent diabetics. J Periodontol.

[5] Mashimo PA, Yamamoto Y, Slots J, Park BH, Genco RJ (1983). The periodontal microflora of juvenile diabetics. Culture, immunofluorescence, and serum antibody studies. J Periodontol.

[6] Casarin RC, Duarte PM, Santos VR, Lima JA, Gagnon G, Casati MZ, Gonçalves RB (2010). Influence of glycemic control on Epstein-Bar and Cytomegalovirus infection in periodontal pocket of type 2 diabetic subjects. Arch Oral Biol.

[7] Makiura N, Ojima M, Kou Y, Furuta N, Okahashi N, Shizukuishi S, Amano A (2008). Relationship of Porphyromonas gingivalis with glycemic level in patients with type 2 diabetes following periodontal treatment. Oral Microbiol Immunol.

[8] Shikarkhane V, Dodwad V, Patankar SA, Pharne P, Bhosale N, Patankar A (2024). Comparative evaluation of mogibacterium timidum in the subgingival plaque of periodontally healthy and chronic periodontitis patients: a real-time polymerase chain reaction (PCR) study. Cureus.

[9] van Pelt-Verkuil E, Van Belkum A, Hays Hays Principles and Technical Aspects of PCR Amplification. https://citations.springernature.com/book?doi=10.1007/978-1-4020-6241-4.

[10] Hashimura T, Sato M, Hoshino E (2001). Detection of Slackia exigua, Mogibacterium timidum and Eubacterium saphenum from pulpal and periradicular samples using the Polymerase Chain Reaction (PCR) method. Int Endod J.

[11] Holdeman LV, Cato EP, Burmeister JA, Moore WE (1980). Descriptions of Eubacterium timidum sp. nov., Eubacterium brachy sp. nov., and Eubacterium nodatum sp. nov. isolated from human periodontitis. Int J Syst Evol Microbiol.

[12] Emrich LJ, Shlossman M, Genco RJ (1991). Periodontal disease in non-insulin-dependent diabetes mellitus. J Periodontol.

[13] Moore PA, Weyant RJ, Mongelluzzo MB (1998). Type 1 diabetes mellitus and oral health: assessment of tooth loss and edentulism. J Public Health Dent.

[14] Hill GB, Ayers OM, Kohan AP (1987). Characteristics and sites of infection of Eubacterium nodatum, Eubacterium timidum, Eubacterium brachy, and other asaccharolytic eubacteria. J Clin Microbiol.

[15] Takahashi K, Nishimura F, Kurihara M, Iwamoto Y, Takashiba S, Miyata T, Murayama Y (2001). Subgingival microflora and antibody responses against periodontal bacteria of young Japanese patients with type 1 diabetes mellitus. J Int Acad Periodontol.

[16] Taylor GW, Burt BA, Becker MP, Genco RJ, Shlossman M (1998). Glycemic control and alveolar bone loss progression in type 2 diabetes. Ann Periodontol.

[17] Borgnakke WS, Genco RJ (2003). Periodontal disease and diabetes mellitus. International Textbook of Diabetes Mellitus.

[18] Hemalatha VT, Manigandan T, Sarumathi T, Amudhan A (2013). Dental considerations in pregnancy-a critical review on the oral care. J Clin Diagn Res.

[19] Kim EK, Lee SG, Choi YH, Won KC, Moon JS, Merchant AT, Lee HK (2013). Association between diabetes-related factors and clinical periodontal parameters in type-2 diabetes mellitus. BMC Oral Health.

[20] Casarin RC, Saito D, Santos VR, Pimentel SP, Duarte PM, Casati MZ, Gonçalves RB (2012). Detection of Mogibacterium timidum in subgingival biofilm of aggressive and non-diabetic and diabetic chronic periodontitis patients. Braz J Microbiol.

[21] Engebretson SP, Hey-Hadavi J, Ehrhardt FJ, Hsu D, Celenti RS, Grbic JT, Lamster IB (2004). Gingival crevicular fluid levels of interleukin-1beta and glycemic control in patients with chronic periodontitis and type 2 diabetes. J Periodontol.

[22] Safkan-Seppälä B, Sorsa T, Tervahartiala T, Beklen A, Konttinen YT (2006). Collagenases in gingival crevicular fluid in type 1 diabetes mellitus. J Periodontol.

